# Supplementary material for: Green Roof Substrate Microbes Compose a Core Community of Stress-Tolerant Taxa
Source: Microorganisms. 2024 Jun 21;12(7):1261. doi: 10.3390/microorganisms12071261 (PMC11279297; doi:10.3390/microorganisms12071261)
Supplement: Supplementary file 1 [file microorganisms-12-01261-s001.zip › microorganisms-2961472-supplementary.pdf]

microorganisms

Supplementary Materials

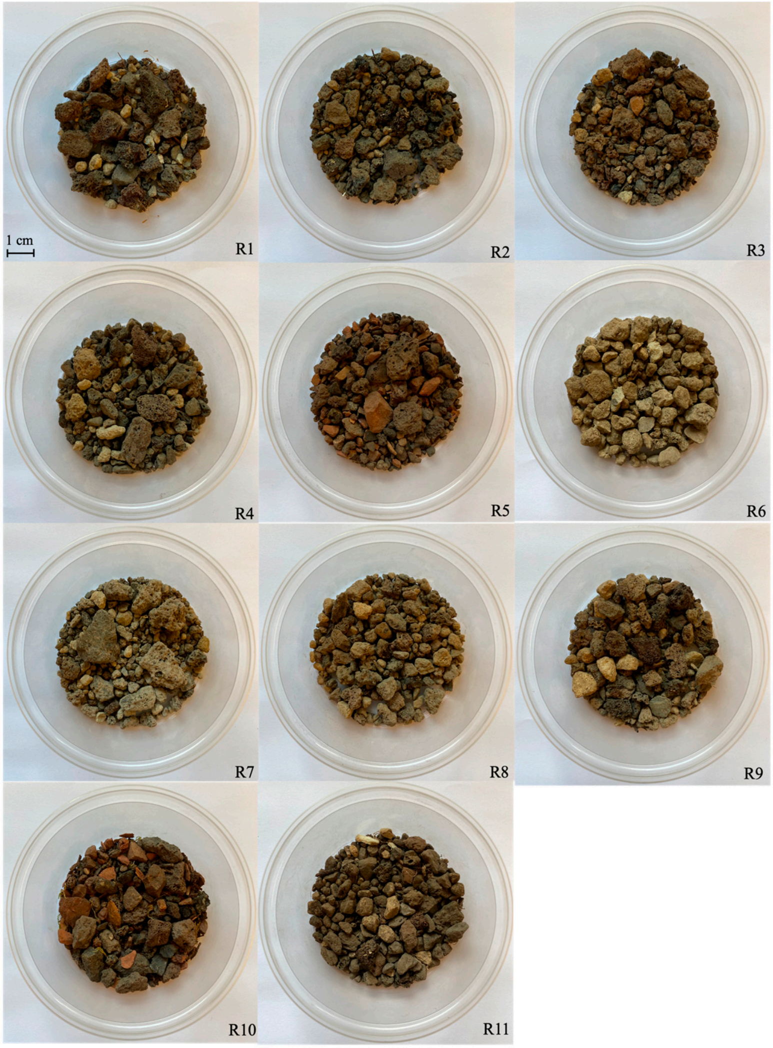

Figure S1. An overview of the substrate textures from every investigated green roof.

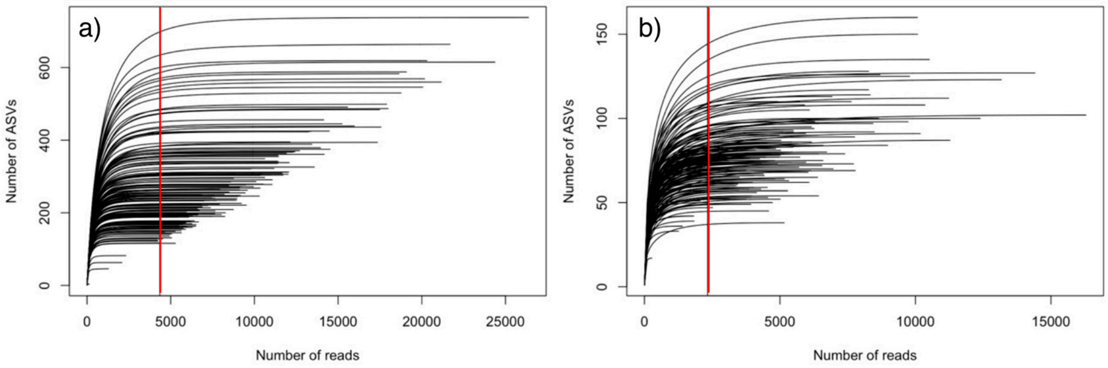

Figure S2. Rarefaction curves. Prokaryotic samples are rarefied to 4,352 reads (a). Fungal samples are rarefied to 2,331 reads (b).

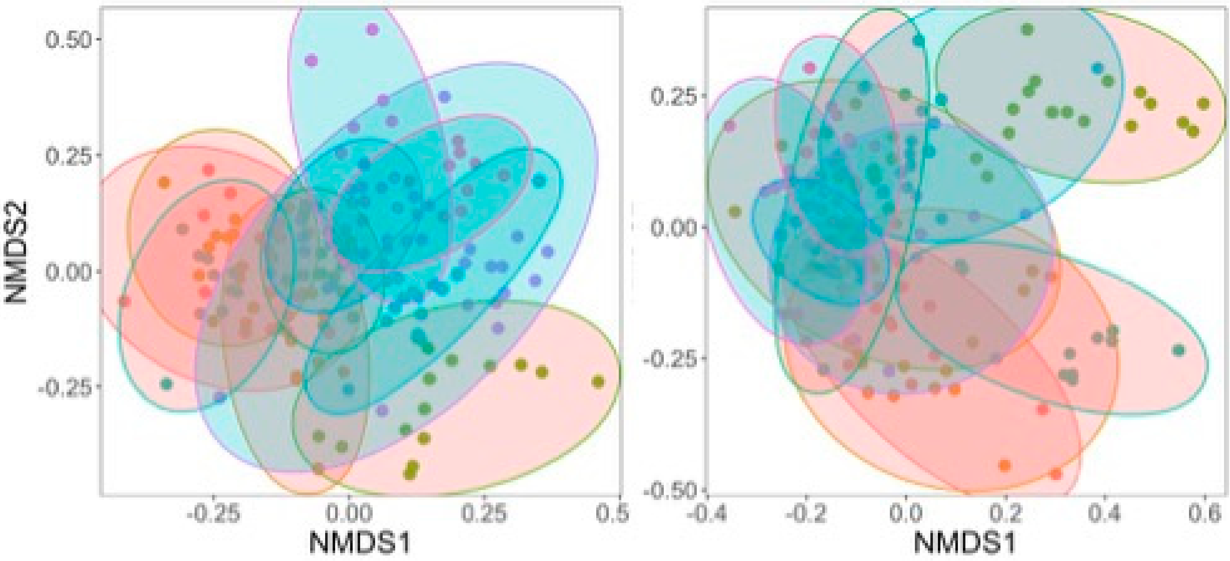

Figure S3. Prokaryotic (a) and fungal (b) beta-diversity. Nonmetric multidimensional scaling (NMDS) plots (based on Bray-Curtis distance similarity matrices) showing beta-diversity of green roof substrate microbes. Prior to ordination, prokaryotic/fungal samples are rarefied to 4,352/2,331 reads. Orange ellipses: *Sedum*-herbs-grasses roofs; blue ellipses: *Sedum*-moss roofs. Many extensive green roofs have distinct prokaryotic or fungal community compositions, even roofs within the same type of vegetation.

Table S1. A list of the plant species that are identified on every green roof during four occasions in the growth season (May, June, August and September 2019). An x denotes the presence of the respective species.

Family	Species	R1	R2	R3	R4	R5	R6	R7	R8	R9	R10	R11
Alliaceae	*Allium schoenoprasum*	x					x	x				x
Alliaceae	*Allium sphaerocephalon*											x
Amaranthaceae	*Chenopodium album*					x					x	
Amaranthaceae	*Chenopodium polyspermum*			x								
Apiaceae	*Daucus carota*										x	
Asteraceae	*Achillea millefolium*						x					
Asteraceae	*Anthemis tinctoria*	x		x								
Asteraceae	*Calendula arvensis*	x										
Asteraceae	*Cirsium dissectum*			x								
Asteraceae	*Conyza canadensis*						x					
Asteraceae	*Conyza sumatrensis*			x								
Asteraceae	*Crepis capillaris*		x					x				
Asteraceae	*Erigeron canadensis*	x	x	x	x	x		x	x	x	x	x

Asteraceae	*Galinsoga quadriradiata*	x				x	x					
Asteraceae	*Gnaphalium luteoalbum*	x										x
Asteraceae	*Hypochaeris radicata*	x		x	x	x	x	x	x	x		
Asteraceae	*Jacobaea vulgaris*											x
Asteraceae	*Lactuca serriola*			x								x
Asteraceae	*Mycelis muralis*							x		x		
Asteraceae	*Picris hieracioides*		x									
Asteraceae	*Pilosella officinarum*	x		x		x						
Asteraceae	*Senecio inaequidens*		x		x		x	x	x	x	x	x
Asteraceae	*Senecio vulgaris*	x		x	x	x	x	x	x	x	x	x
Asteraceae	*Sonchus asper*					x		x	x			x
Asteraceae	*Sonchus oleraceus*	x	x	x	x	x	x	x	x	x	x	x
Asteraceae	*Taraxacum palustre*		x									
Asteraceae	*Tragopogon dubius*										x	
Boraginaceae	*Anchusa officinalis*			x								
Boraginaceae	*Echium vulgare*					x		x		x		
Boraginaceae	*Myosotis arvensis*	x										
Brassicaceae	*Alyssum alyssoides*	x				x						
Brassicaceae	*Barbarea vulgaris*	x	x									
Brassicaceae	*Capsella bursa-pastoris*										x	
Caryophyllaceae	*Arenaria serpyllifolia*	x						x			x	
Caryophyllaceae	*Cerastium glomeratum*						x		x	x		
Caryophyllaceae	*Dianthus armeria*	x		x								
Caryophyllaceae	*Dianthus carthusianorum*	x						x				x
Caryophyllaceae	*Dianthus deltoides*			x								
Caryophyllaceae	*Petrorhagia prolifera*	x		x		x					x	
Caryophyllaceae	*Silene vulgaris*	x		x		x						
Caryophyllaceae	*Stellaria media*					x						
Crassulaceae	*Sedum acre*	x				x			x	x		

Crassulaceae	*Sedum album*	x	x		x	x	x	x	x	x	x	x
Crassulaceae	*Sedum forsterianum*											x
Crassulaceae	*Sedum hispanicum*	x	x	x	x	x	x			x	x	x
Crassulaceae	*Sedum hybridum*	x	x	x	x	x	x	x	x	x		x
Crassulaceae	*Sedum kamtschaticum*	x	x		x			x				x
Crassulaceae	*Sedum oregonense*											x
Crassulaceae	*Sedum rupestre*	x	x	x		x		x	x		x	
Crassulaceae	*Sedum sexangulare*	x	x	x		x	x	x	x	x	x	x
Crassulaceae	*Sedum spurium*	x	x	x	x	x	x	x	x	x	x	x
Euphorbiaceae	*Euphorbia cyparissias*	x		x								
Euphorbiaceae	*Euphorbia peplus*			x	x	x						
Fabaceae	*Lupinus polyphyllus*	x										
Fabaceae	*Medicago minima*	x										
Fabaceae	*Melilotus albus*				x			x				
Fabaceae	*Trifolium arvense*		x	x	x					x	x	
Fabaceae	*Trifolium dubium*		x	x	x	x		x			x	
Fabaceae	*Trifolium repens*				x							
Geraniaceae	*Erodium cicutarium*	x		x	x							x
Geraniaceae	*Geranium molle*				x	x			x		x	x
Geraniaceae	*Geranium purpureum*	x				x		x			x	
Geraniaceae	*Geranium pusillum*					x		x				x
Geraniaceae	*Geranium robertianum*			x	x							
Hypericaceae	*Hypericum perforatum*		x				x	x				
Juncaceae	*Juncus effusus*			x								
Lamiaceae	*Clinopodium acinos*					x						
Lamiaceae	*Origanum vulgare*	x		x		x						
Lamiaceae	*Prunella vulgaris*			x								
Lamiaceae	*Thymus pulegioides*			x		x	x					
Lamiaceae	*Thymus serpyllum*			x								

Lamiaceae	*Thymus vulgaris*							x				
Leguminosae	*Vicia hirsuta*				x	x	x	x	x		x	
Leguminosae	*Vicia sativa*										x	
Leguminosae	*Vicia tetrasperma*							x				
Linaceae	*Linum austriacum*	x										
Montiaceae	*Claytonia perfoliata*								x			
Onagraceae	*Epilobium tetragonum*						x					x
Papaveraceae	*Papaver argemone*	x				x						
Plantaginaceae	*Chaenorhinum minus*					x	x	x				
Plantaginaceae	*Cymbalaria muralis*			x								
Plantaginaceae	*Linaria vulgaris*			x		x	x				x	
Plantaginaceae	*Plantago major*			x								
Plantaginaceae	*Veronica arvensis*	x			x	x	x	x				x
Poaceae	*Bromus sterilis*		x									
Poaceae	*Bromus tectorum*					x						
Poaceae	*Calamagrostis epigejos*		x									
Poaceae	*Cynodon dactylon*					x						
Poaceae	*Festuca filiformis*					x	x	x	x		x	x
Poaceae	*Festuca rubra*	x	x	x	x	x	x					
Poaceae	*Holcus lanatus*	x	x				x					
Poaceae	*Hordeum vulgare*										x	
Poaceae	*Lolium perenne*		x									
Poaceae	*Molinia caerulea*									x		
Poaceae	*Phalaris arundinacea*							x				
Poaceae	*Poa annua*						x					x
Poaceae	*Vulpia myuros*										x	
Polygonaceae	*Fagopyrum esculentum*			x								
Polygonaceae	*Rumex acetosella*	x										
Ranunculaceae	*Nigella arvensis*					x						

Rosaceae	*Potentilla erecta*		x		x	x		
Rosaceae	*Sanguisorba minor*				x	x		
Rubiaceae	*Galium verum*	x						
Scrophulariaceae	*Verbascum densiflorum*				x			
Scrophulariaceae	*Verbascum thapsus*						x	
Solanaceae	*Solanum americanum*				x		x	
Verbenaceae	*Verbena bonariensis*							
Violaceae	*Viola arvensis*			x		x		x
